# Comparison of Three Methods of Measuring Residual Stresses in Welded Joints of High-Strength Steel S960QL

**DOI:** 10.3390/ma18050950

**Published:** 2025-02-21

**Authors:** Mirza Manjgo, Gorazd Lojen, Nenad Gubeljak, Blaž Karpe, Tomaž Vuherer

**Affiliations:** 1 Faculty of Mechanical Engineering, University of Maribor, Smetanova Ulica 17, 2000 Maribor, Slovenia; mirza.manjgo@student.um.si (M.M.); gorazd.lojen@um.si (G.L.); nenad.gubeljak@um.si (N.G.); 2 Faculty of Natural Science and Technology, University of Ljubljana, Aškerčeva Cesta 12, 1000 Ljubljana, Slovenia; blaz.karpe@ntf.uni-lj.si

**Keywords:** weld joint, high-strength steel, residual stress, magnetic method, X-ray diffraction method, hole drilling method

## Abstract

The influence of residual stresses as a result of the welding process in the overall stress state of the weld joint is of great importance because they significantly affect the creation and growth of cracks, the occurrence of brittle fracture, and material fatigue. Previous experiences indicate that it would be necessary to provide an assessment of the deformation and stress state in the critical zones of the weld joints using a suitable test method, which will not endanger the structural integrity of the tested places. There are different methods for measurement of residual stress in welded constructions: destructive, semi-destructive and non-destructive. To choose one method over another, it is necessary to take into account the advantages and limitations of these techniques for practical application. This paper considers and analyzes the residual stresses in the welded joint of high-strength steel S960QL. MAG welding was performed by a robot. Three methods were used to measure the residual stresses: the magnetic method (MAS), the X-ray diffraction method (XRD), and the hole drilling method (HD). By all three methods, the highest residual stresses were measured in the weld metal and in the heat-affected zones. Nevertheless, the measured values differed considerably. The differences can be contributed to (a) the kind of stress that the individual method measures, (b) to the volume of material from which each method captures the signal and averages it, and (c) to the different sensitivities of the applied methods to coarse-grained microstructure and microstructural gradients.

## 1. Introduction

**Figure 1:**
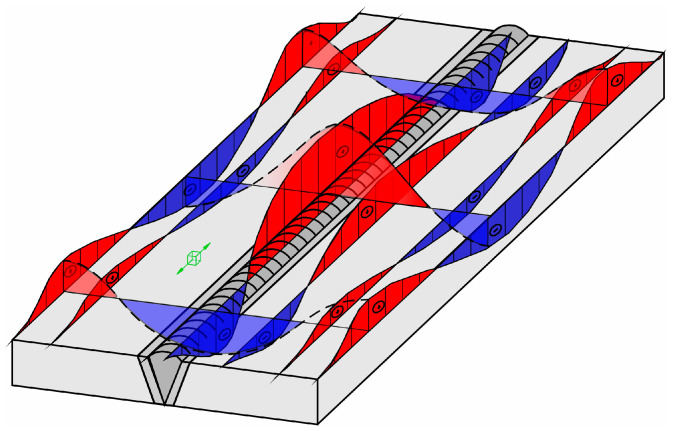
Longitudinal residual stresses in welded austenitic stainless steel, where no phase transformations in the solid state occur; red: tensile stress, blue: compressive stress.

**Figure 2:**
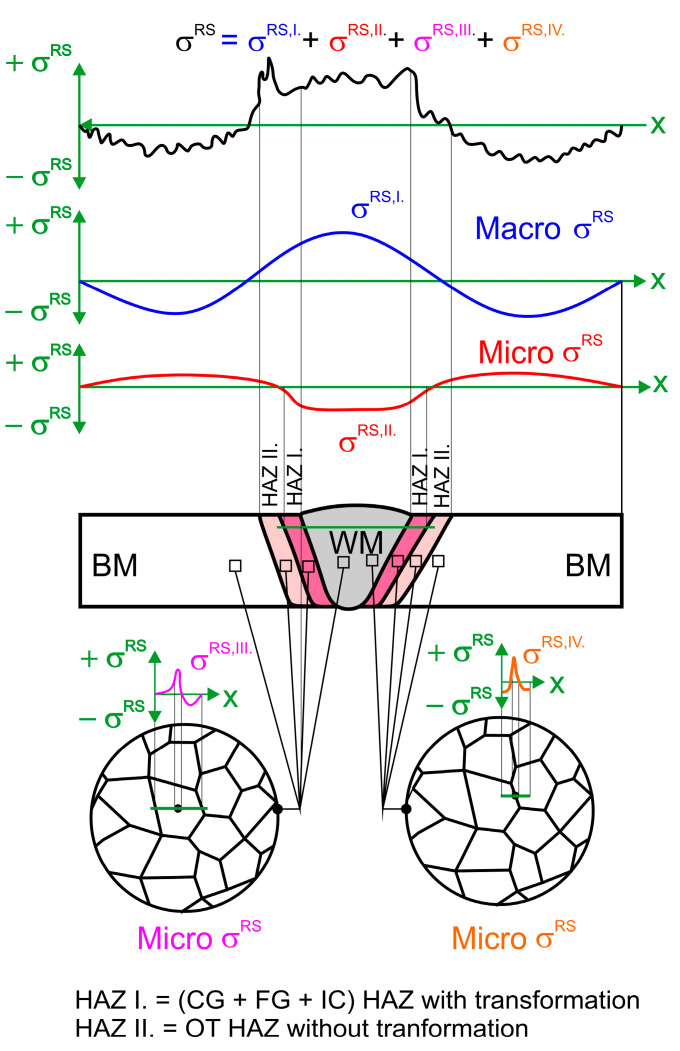
Welded construction steel, subjected to phase transformations in the solid state. Total residual stress distribution along several crystal grains of a polycrystalline material and their partitioning in type I (*σ*^RS,I^.—macro residual stresses), type II (*σ*^RS,II^.—homogeneous micro residual stresses) and type III and type IV (*σ*^RS,III^.—inhomogeneous micro residual stresses inside grains and *σ*^RS,IV^.—inhomogeneous micro residual stresses between grains and grain boundary).

The quenched and tempered group is the most frequently used high-strength structural steel category, and S960QL is the highest grade according to the EN 10025-6 standard, although nowadays higher-strength grades are available but not yet classified using the standard [1]. The outstanding strength properties can be particularly beneficial in the case of mobile structures like vehicles, cranes, and excavators, where due to reduced dead weight, significant energy savings can be achieved and/or workloads can be increased [2]. In the production of such structures, welding is among the most important joining techniques. The S960QL steel is readily weldable. Different processes are applicable: electron beam and laser welding [3], TIG [4,5], SAW [6], and, in industrial production, most often the GMAW process [1,7,8,9]. Nevertheless, welding is more complicated than in the case of mild steels due to the risk of cold cracking and the decrease in toughness and strength properties in heat-affected zones [2,7]. In addition, the appearance of residual stress (RS) caused by restrained thermal expansion and shrinkage due to rapid local heating and subsequent cooling of the workpiece can neither be avoided nor, in the case of S960QL, be reduced by post weld heat treatment as effectively as in low-strength structural steels, as according to the producers (e.g., SSAB [10] and ThyssenKrupp [11]) stress relieve temperature must not exceed 550–560 °C. Due to residual stresses, stress levels in materials can be increased. Loads resulting from superposition of workload and residual stress can easily exceed the material’s fatigue limit or even reach the yield strength. Thereby, residual stresses reduce the fatigue life of the welded joints [12]. Even moderate residual stresses (<50% of the yield strength) have a huge impact on the fatigue performance [13]. Therefore, as accurate knowledge as possible of residual stress-fields in the welded component is essential for prediction of its life span. Nevertheless, assessment of residual stress is often a quite difficult task. With respect to the size of the affected area, four types of residual stresses can be defined: stresses of type I, type II, type III, and type IV [14,15,16,17]. The type I residual stresses or macro residual stresses span over several crystal grains and are balanced throughout the volume. They are called macro stresses, because a change in residual stresses of type I results in dimension change. Residual stresses of the type II or homogeneous micro residual stresses are almost homogeneous inside a microscopically small area (inside a single grain or a part of a grain) and are balanced inside of a sufficiently large group of grains. In weld joints, such regions are weld metal and HAZ, where phase transformations *α* to *γ* during heating and *γ* to *α* during cooling took place—WM + HAZ I (coarse-grained HAZ, fine-grained HAZ and inter-critical HAZ). Type III and type IV residual stresses or inhomogeneous micro residual stresses are inhomogeneous, and limited to small sub microscopic regions, i.e., several interatomic distances inside of individual grains or between crystal grain and its grain boundary. Type III residual stresses occur inside crystal grains due to presence of crystal defects (i.e., precipitates during aging, inclusions inside crystals grains what can result in higher strength of material), while type IV residual stresses occur between crystal grain and grain boundary (i.e., carbides on grain boundary to prevent creep). Total residual stresses are, in general, superimposed stresses of all four types. For a simpler case, when no phase transformations in the solid state take place (austenitic stainless steel), an example of longitudinal stresses is presented in Figure 1. An example of a black construction steel, where, in addition to solidification and grain coarsening, phase transformations in the solid state also take place, is presented in Figure 2.

Over time, numerous different methods for measuring residual stresses have been developed. They differ not only in the accuracy and reliability of the measurement, but also the size and availability of equipment, physical principle of the measurement, sampling area/volume, and consequently in the field of application. With respect to the influence on the test piece, they can be divided into three major groups: destructive, semi-destructive and non-destructive methods [15,16,18]. Destructive and semi-destructive methods rely on measurement of deformations due to the relaxation of residual stresses after some material is removed from the test piece. Sectioning and contour methods are the most common destructive techniques. Hole drilling (HD), incremental hole drilling (IHD), dip hole drilling (DHD) and ring core are the commonly used semi-destructive techniques. Non-destructive methods include the X-ray diffraction (XRD), synchrotron diffraction, neutron diffraction, Raman spectrometry, ultrasonic methods, and magnetic methods. If a destructive method is applied, the test piece is destroyed. Therefore, for industrial use, non-destructive methods are more convenient. Very often also semi-destructive methods like HD and IHD are applicable, because their impact on the integrity of the test piece is small enough to be neglected, tolerated, or easily repaired [19]. For on-site testing, it is further important that portable equipment is available, like for the HD, XRD, and or magnetic methods.

Over the last two decades, several reviews have been made, identifying the advantages and disadvantages of numerous methods. Most comprehensive were reviews made by Kandil et al. [20], Rossini et al. [16], and Guo et al. [21]. They established that the most popular method was the HD method followed by the XRD method. Considering numerous other reports, this is not surprising. The HD method is the most frequently used method because it is relatively simple, quick and precise [16,22,23,24,25], it is of high theoretical maturity [21], and it is standardized in ASTM E837 [16,26,27], which is very convenient for practical use. Consequently, the method was often used to validate results of other measuring methods like XRD [28], or novel methods under development [19,22,29]. Among the disadvantages of HD and IHD, following should not be neglected: Only the macroscopic residual stresses of the type I can be determined, and the accuracy suffers in textured and inhomogeneous anisotropic microstructures [22]. Ultra-high-speed drilling is necessary to introduce as little as possible stress by drilling, and if stresses are not uniform through the depth, drilling should be incremental (IHD technique) [30]. Furthermore, performance of the HD method is directly related to the quality of the strain gauge rosette and its installation, wiring, strain measuring instrument, the quality of the drilling machine and cutter, details of experimental procedure followed by the operator, and it is sensitive to stress gradients [31].

The XRD method also has many advantages. It is relatively cost-effective and widely available with portable and robotic diffractometers [27], exhibits high accuracy [21,24], and is standardized in standards EN 15305 [32] and ASTM E2860 [33]. X-rays can penetrate non-crystalline surface layers like corrosion protection maintaining reasonable accuracy [28]. Nevertheless, the presence of surface coatings, oxide layers, etc., can result in reduction in the intensity of diffracted X-rays, so, to assure maximum accuracy, surface should be cleaned, which is usually performed almost completely non-destructively by electropolishing [34]. As the intensity of the incident X-rays decreases exponentially with the depth below the surface, XRD strain measurement is limited to very shallow surface layers [35]. Furthermore, XRD is applicable only on crystalline materials [16], whereat coarse-grained size diminishes reliability and can even make the results unusable [36]. Therefore, the grain size of the workpiece should not exceed 100 μm, preferably it should be less than 30 μm [21].

In several reports, results obtained by HD and XRD were directly compared. Baig et al. [37] reported good agreement of results obtained with HD and XRD on welded samples. Jo et al. [38] measured residual stresses in spot welded high-strength steel. Results obtained by the HD method were similar to those obtained by XRD. Yang et al. [28] measured residual stress in welds in ship building steel and reported that trend and magnitude of the XRD and HD results were similar.

Considering that especially on welded parts and constructions, often dozens of measurements are necessary, neither the HD nor XRD method is fast. Each XRD measurement takes ca. 15 min and HD measurement ca. 1 h. Therefore, despite generally good results and practical applicability of the HD and XRD methods, there is steel room for other methods, preferably faster ones, especially for industrial use.

Suitable candidates can be found among the ultrasonic and magnetic methods. They are easy to use, fast, and low-cost methods but they have low resolution [Rossini-2012-14]. At present, the accuracy of general equipment for ultrasonic residual stress measurement can reach approximately ±30 MPa. Among the magnetic methods, two methods based on magnetoelastic effect are relatively widespread. Barkhausen emission (BE, also known as BNE or MBE) which measures electromagnetic pulses (Barkhausen noise) caused by stress-induced irreversible domain wall movements under an applied magnetic field. BE is particularly sensitive to the microstructure and mechanical properties of the material and is sensitive to surface quality—preparation [39]. The other is magnetic anisotropy system (MAS) which is based on stress-induced magnetic anisotropy and measures magnetic permeability (the result of measurement is the subtraction of principal stresses *σ*_1_–*σ*_2_).

Probe consists of two solenoids that are orthogonally positioned. Solenoids have cores made of magnetic material, in order to ensure higher magnetic excitation. The device measures magnetic permeability in the plain along the chosen direction due to the core orientation from one pole to another. Probe position (orientation), which yields maximum stress, corresponds to maximum principal stress *σ*_1_. Principal stress *σ*_2_ stress is obtained when probe is rotated for 90°. Magnetic permeability is proportional to the deformation which is itself proportional below 0.8 yield stress. If this value is exceeded, the relation follows the so-called “S” curve and nonlinear behavior, which is determined with calibration, should be taken into account [40].

For MAS, the surface of the material requires no special treatment except that it must be flat.

In principle, MAS is accurate, and uncertainties are in order of 1 MPa, while for harder materials such as fully pearlitic or martensitic steels the uncertainties become larger, typically a few tens of MPa [41]. Additionally, textured microstructures increase uncertainty. Bešević et al. [40] applied this method on cold formed steel and established accuracy within ±40 MPa. To increase accuracy, it is critical to obtain calibration curves with calibration samples similar to the actual component to be tested [20,21,40,41]. For welded test pieces this means, that calibration samples must consist of weld, HAZ and base metals, and they must be welded with the same process and welding parameters as the real test piece [30]. Authors considered MAS as a comparative method [40,42], as a possibility to reduce measurement costs by combining MAS and HD—first, it is necessary to perform magnetic method measurements on as many points as possible, and then perform a check of residual stresses on some measurement points with rosette strain gauge method.

No comparisons of results obtained with MAS and XRD on the same test piece or direct comparison of results obtained with more than two methods could be found in the open literature.

Due to the small allowable heat input, steep microstructural gradients can be expected in S960QL welds, which reduce the reliability of the results for most residual stress measurement methods. Consequently, the importance of comparing results obtained by different methods increases.

To date, the open literature reported residual stress measuring on S960QL welded components almost exclusively with XRD [3,4,9,12,43,44], and only in two cases were comparative measurements performed by hole drilling [9] and neutron diffraction [4].

The focus of this work was on the performance of three different methods for measuring residual stress on welded S960QL plate where non-uniform residual stresses were present: the MAS, XRD, and incremental HD methods.

The purpose of this paper was not to compare the accuracy of the individual method or statistical evaluation of the results obtained by any of them. Reliably statistical analysis is only possible if the test pieces are homogeneous with respect to microstructure and stress fields, so that results obtained in several points can be compared. Unfortunately, welded test pieces are markedly inhomogeneous with respect to stress fields, as shown in Figure 1 and Figure 2, and with respect to microstructure. Consequently, results obtained in different measuring points cannot be compared. Only results obtained at the same measuring point can be compared, and even then only to a certain extent, because the applied methods have different sensitivities to stress gradients and microstructure, cover different volumes of material, and therefore measure different types of residual stress.

## 2. Materials and Methods

### 2.1. Material and Welding

We used 15 mm-thick steel plates made of high-strength S960QL steel according to EN 10025-6:2019+A1:2023 [45] as the base material. The chemical composition of the steel is given in Table 1, and the basic mechanical properties in Table 2.

**Figure 3:**
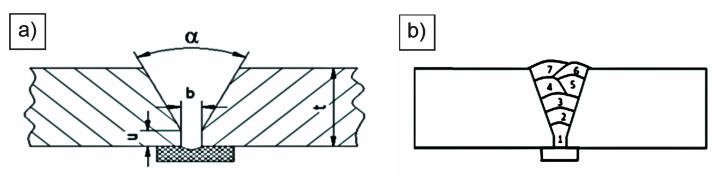
(**a**) Geometry of the weld groove; (**b**) sequence of weld passes.

Two equally sized plates, 15 mm × 160 mm × 700 mm, were welded with MAG process with a solid metal wire (135 according to EN ISO 4063:23 [46]). The geometry of the single-V butt weld was as follows: plate thickness *t* = 15 mm groove angle *α* = 50°, root opening *b* = 3 mm, and root face *u* = 1 mm, as shown in Figure 3a. The sequence of passes is shown in Figure 3b.

The filler material was a solid wire *ϕ* 1.0 mm BÖHLER X 90-IG (G 89 6 M21 Mn4Ni2CrMo according to EN ISO 16834-A [47]). This type of wire is designed for welding of high-strength steels including S960QL. The guaranteed minimum yield strength of the wire is 890 MPa, which is slightly lower than that of the base metal, whereat the ultimate tensile strength (UTS) is 940–1180 MPa, which matches the requirements for the base metal’s UTS very well. The brand of the wire was chosen based on availability from our supplier. Chemical composition is given in Table 3.

Shielding gas was M21 according to EN ISO-14175:2008 [EN-ISO-14175:2008] containing 82% Ar + 18% CO_2_. High argon content reduced spraying and assured low level of oxidation. The CO_2_ stabilized the electric arc and increased penetration. Preheating temperature was calculated according to EN 1011-2:2001 [48], Annex C, Method B.

The welding was performed in the industrial environment by a robot OTC FD-V8L (OTC Daihen Europe, Mönchengladbach, Germany) and welding machine WelBee P500L (Daihen Varstroj d.d., Lendava, Slovenia). The welding parameters are given in Table 4.

### 2.2. The Tensile Test

**Figure 4:**
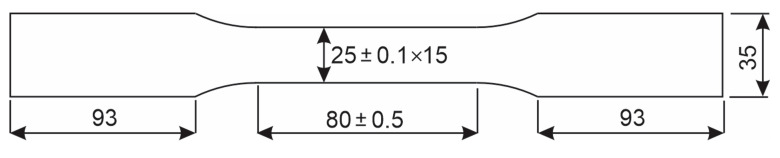
Geometry of tensile specimens.

The tensile probe is a mechanical test, in which the specimen is subjected to uniaxial loading, which gradually increases. The test was performed according to standard EN ISO 6892-1:2019 by using method B [49] on the servo hydraulic Amsler 559/594 universal testing machine (Amsler Prüfmaschinen A.G., Merishausen, Switzerland—now Amsler Prüfsysteme, Neftenbach, Switzerland). Two specimens were taken out from the welded plate so that the weld was in the middle of the gauge length. The fracture was expected to occur in the zone of the welded joint. The geometry of welded specimens is shown in Figure 4, and corresponds to the standard EN ISO 4136:2022 (destructive tests on welds in metallic materials—transverse tensile test) [50], which requires a minimum width of 25 mm and a minimum parallel length greater or equal to the width of the weld +60 mm.

To test the base metal, standard flat specimens according to EN ISO 6892-1:2019 were used, with a cross-section *S*_0_ = 25 mm × 15 mm, and a gauge length $L_{0}=5.65\times \sqrt{S_{0}}$.

### 2.3. Residual Stress Measurement

**Figure 5:**
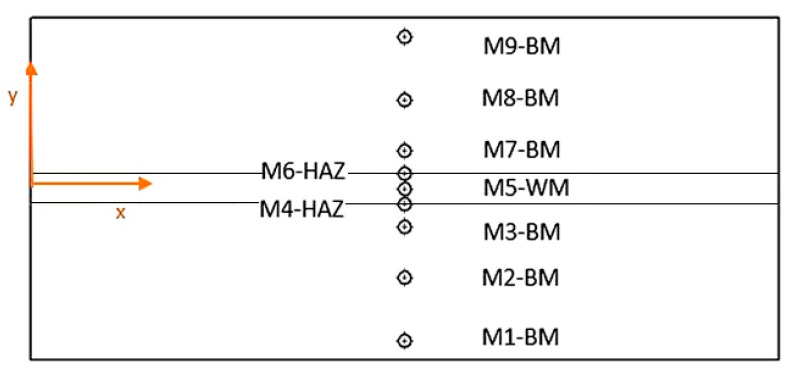
Coordinates of measuring points (see Table 5).

Prior to measurements of residual stresses (RSs), the plate was demagnetized. RSs were measured on predefined locations along a line perpendicular to the welding direction, as shown in Figure 5. At each location, RSs were measured first by MAS, then by XRD, and finally by HD. The coordinates of measuring points are given in Table 5.

Some difficulties when measuring in the weld and HAZ must be expected. On the one hand, volumes covered by MAS and probably also by XRD are too large to give results only for HAZ material. In particular, MAS also covered significant portions of weld metal and base metal. On the other hand, all applied methods require a flat surface. Therefore, the weld reinforcement had to be removed to make measurements possible, which certainly affected the residual stresses. To minimize the influences of surface preparation whenever possible, the measurements were carried out in the following order:

(1) The magnetic method (no surface damage except in in the weld metal and partially in HAZ).
(2) The XRD method (minimal surface damage due to electropolishing of the surface with NaCl).
(3) The hole drilling method (drilling a ***ϕ*** 1.72 mm hole to a depth of 1 mm, which prevented any repetition of measurements at the same measuring point).

#### 2.3.1. The Magnetic Method—MAS

**Figure 6:**
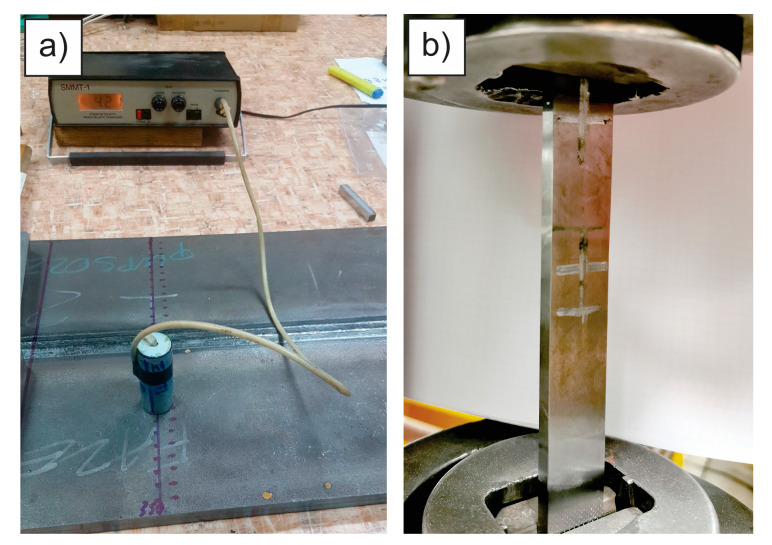
(**a**) SMMT-1 was used for measurement; (**b**) a tensile test specimen was used for calibration.

A SMMT-1 device was used (E. O. Paton Electric Welding Institute, Kiev, Ukraine), as shown in Figure 6a. The probe measures the average voltage value of an area with a diameter of 25 mm to a depth up to 0.5 mm. Calibration was performed on a universal Serservo hydraulic testing machine Amsler 559/594 (Amsler Prüfmaschinen A.G., Merishausen, Switzerland—now Amsler Prüfsysteme, Neftenbach, Switzerland). For determination of the calibration curve, a welded, stress-relieved and demagnetized flat specimens having a cross-section of 25.1 mm × 7.5 mm was used, which was loaded up to *R*p_0.2_, as shown in Figure 6b.

At a regular tensile test, narrower gauge length and wider gripping sections (like shown in Figure 4 ensure that the test piece does not prematurely break at the jaw edge due to notching effects before the capacity for further plastic deformation in the gauge length has been fully exhausted. However, when calibrating a magnetic probe, the loads do not exceed the yield stress, and therefore a simpler geometry (Figure 6b) is also adequate. As the magnetic probe diameter was 25 mm, the width of the test piece had to be at least 25 mm.

**Figure 7:**
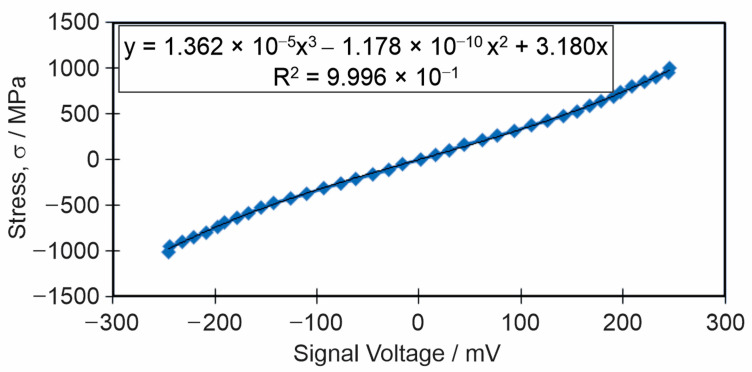
Calibration curve of stress–signal voltage.

The calibration was carried out in steps of 50 MPa. After each 50 MPa increase in stress, the loading was interrupted. At constant load, after a delay of 20 s, signals (voltage) were read at four different probe positions: at 0° and rotated by 90°, 180° and 270°. The load was then increased by a further 50 MPa, measurements were repeated, etc. The procedure was repeated during unloading, also in steps of 50 MPa. Finally, for each load step, the signal value was calculated as the average of all readings made during the increasing and decreasing load. In this way, two calibration curves were obtained. One for the base metal (Figure 7) and one, very similar to the first one, with the probe positioned in the center of the weld. Due to the size of magnetic probes and small width of HAZ obtaining sufficiently reliable curve for HAZ is practically impossible, so the third calibration curve for HAZ was omitted.

The voltage–stress relation is nonlinear and follows the so-called “S” curve. This is because magnetic permeability is proportional to deformation which is itself proportional only below 0.8 yield stress. If this value is exceeded, the relation becomes nonlinear [40].

In general, no surface preparation is necessary for magnetic measurements. Nevertheless, as the probe requires a flat surface, the face of the weld was flattened with grinding papers up to 600 grit. No preparation was performed elsewhere.

Four measurements were taken at each measurement point and the average value was calculated as recommended by the device producer.

#### 2.3.2. The X-Ray Diffraction Method—XRD

**Figure 8:**
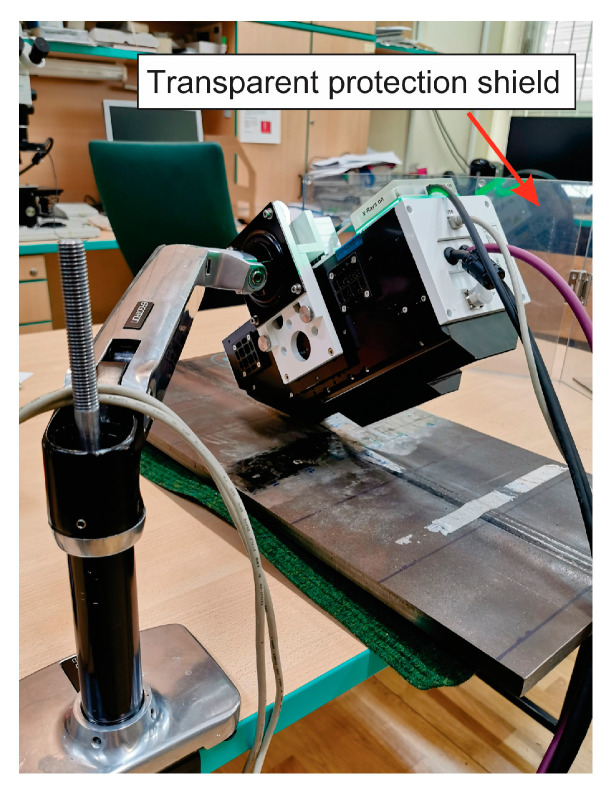
Measuring device PULSTEC U-X360 for the XRD method.

Before measuring, the surface layer was removed by electropolishing. In order to successfully perform electropolishing, it is important to choose the correct electrolyte and electropolishing parameters. The EP3 (Pulstec Industrial Co., Ltd., Hamamatsu, Japan) device was used for electropolishing. The electropolishing anode (+) is a magnet that is placed on the surface of the sample to be electropolished. The cathode (−) represents the electrode of the device EP3. The electrolyte closes the current loop between the electrodes. The depth of electropolishing depends on the condition of the surface and the material and is approximately ~100 μm. Electropolishing was performed on a surface of Ø 5 mm, with a 10% NaCl solution in water with a current of 0.6 A for 3 min, and polishing speed of 40 μm/min. For measurements, a Pulstec U-X360 device was used (Pulstec Industrial Co., Ltd., Hamamatsu, Japan), as shown in Figure 8. It works with a fixed angle of incident X-rays. A circular mask for X-rays (a collimator) limits the irradiated surface area to a diameter of 2 mm. The irradiated surface is marked by a laser pointer which allows precise selection of the analyzed area. All deflected X-rays are collected by a two-dimensional detector during only one exposure, enabling the image of the entire Debye–Scherrer ring where the final value of residual stress in the direction of measuring is automatically determined.

Residual stresses were measured in two directions, parallel and perpendicular to the welding direction. The X-ray diffraction system used automatically makes several hundred readings at each spot and the deviation in a frame of each measurement and finally gives the average value and the deviation. So, the measurement was only carried out once at each measuring point, and the deviations were ±10–17 MPa—except for the measuring point M6, where the deviation was much larger. Therefore, at M6 measurements in both directions were repeated three times, but deviations were always between ±65 MPa and ±72 MPa.

During exposure, a transparent protection shield, supplied with the X-ray device, was used, indicated with a red arrow in Figure 8.

#### 2.3.3. The Hole Drilling Method—HD

**Figure 9:**
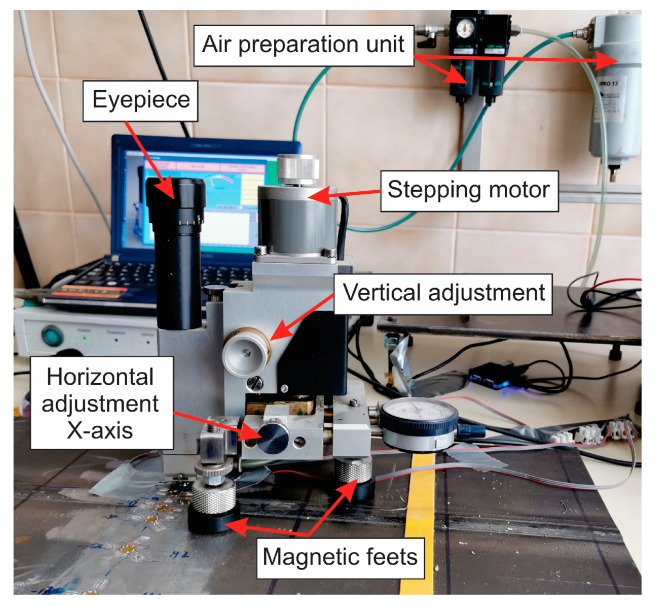
The device MTS3000 RESTAN for centering the drill and drilling a hole at residual stress measurement.

The most widely used and most reliable modern technique for measuring residual stress is the method of “hole drilling”. As the accuracy and reliability of this method have been experimentally proven (the accuracy of this method is ±8%), it is standardized as Standard ASTM E837-20. In this method, the deformation sensor (strain gauge rosette) is first glued to the surface of the test part. For centering the drill and drilling a hole, a MTS3000 RESTAN measurement system was used (SINT Technology S.r.l, Calenzano, Italy), as shown in Figure 9. The depth of drilling was 1.0 mm. Strain gauges—rosettes 1-RY61-1.5/120—were used (Hottinger Baldwin Messtechnik GmbH, Darmstadt, Germany). For data acquisition, a Spider 8 acquisition system was used, and for evaluation, the EVAL 7 software (version 5.3.1) was used (both from SINT Technology s.r.l, Calenzano, Italy).

To attach the rosettes, the surface of every measuring point was first ground with papers up to 600 grit, etched with a Vishay conditioner A for 2 min (Vishay Precision Group, Inc., Malvern, PA, USA), then the Vishay neutralizer 5A was applied, and final cleaning was performed with acetone. To attach the rosettes, the Hottinger two-component adhesive X60 was used.

The mill diameter was 1.72 mm. The feeding pressure for the air turbine was 5 bar, and the rotation speed 400,000 rpm. The drilling rate was 0.2 mm/min, and the drilling was performed in 10 steps. The system’s default setting off acquisition delay is 3 s, but as in our prior experience, longer delays can improve accuracy of results, we selected a longer delay of 5 s, and the drill delay was also 5 s. The baud rate was 115,200.

### 2.4. Optical Microscopy

**Figure 10:**
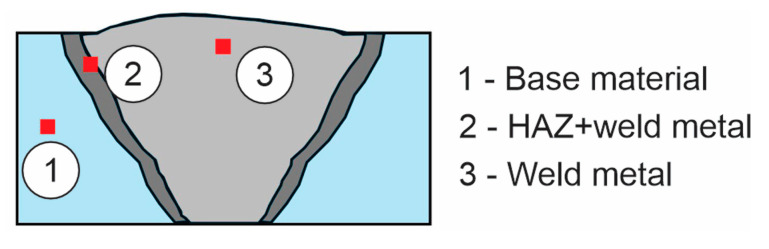
Microstructures in three areas of the weld join.

The microstructures were analyzed in three areas of the weld join, in the weld reinforcement (weld metal, WM), in the heat-affected zone (HAZ) and in the unaffected base material (BM), as shown in Figure 10. A Nikon Epiphot 300 optical microscope (Nikon, Tokio, Japan) equipped with an Olympus DP-12 digital camera (Olympus, Boston, MA, USA) was used.

## 3. Results

### 3.1. Tensile Test Results

The tensile test was performed on two specimens: TT-1 and TT-2. Table 6 shows the results of the tensile test. In both cases, the fracture occurred in the area of base material.

### 3.2. Results of the Magnetic Method

**Figure 11:**
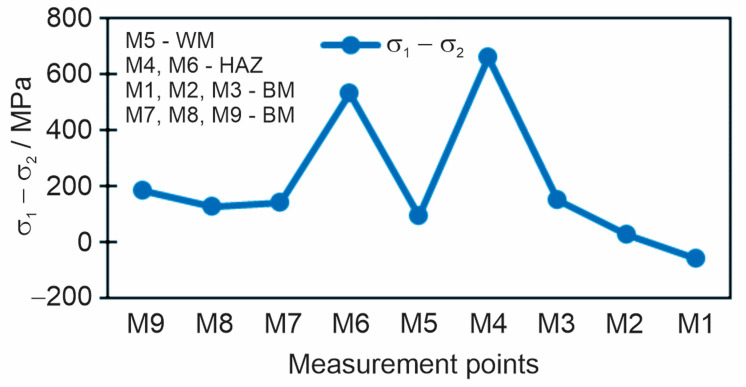
Subtraction of the principal residual stresses *σ*_1_–*σ*_2_, measured by the magnetic method.

Before measuring, the plate was demagnetized. Then, the stresses in the characteristic directions were measured, the longitudinal and the transverse stresses in the different directions (M1, M2, M3, M4). The principal residual stresses in the longitudinal (*σ*_1_) and transversal (*σ*_2_) directions are obtained as the arithmetic mean of two measurements in different directions along with the calibration signal. The subtraction of principal stresses is shown in the diagram in Figure 11. Also, measured and calibrated residual stress values using the magnetic method are given in Table 7.

### 3.3. Results of the X-Ray Diffraction Method

**Figure 12:**
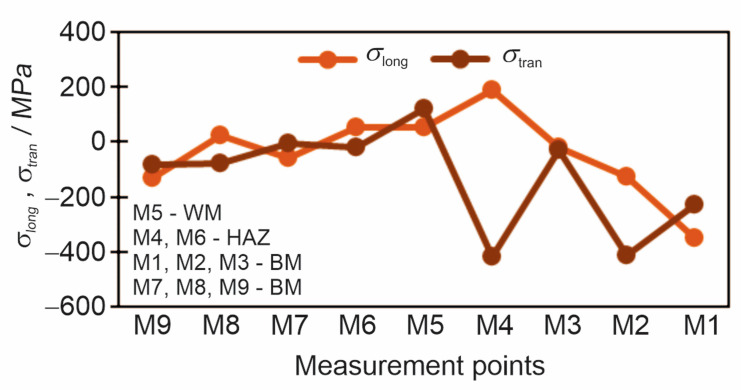
Residual stresses in the longitudinal and transverse directions measured by the X-ray diffraction method.

**Figure 13:**
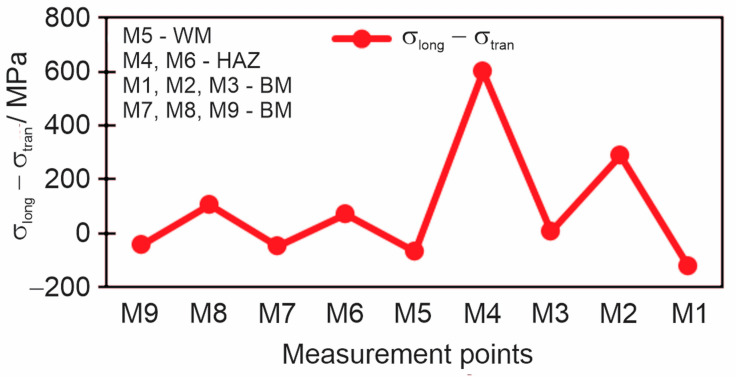
The Subtraction of longitudinal and transverse residual stresses *σ*_long_–*σ*_tran_ measured by X-ray diffraction.

The residual stresses measured by the X-ray diffraction method are in the longitudinal and transverse directions in relation to the welding direction. Diagrams (Figure 12 and Figure 13) and Table 8 show the results obtained by the X-ray diffraction method.

### 3.4. Results of the Hole Drilling Method

**Figure 14:**
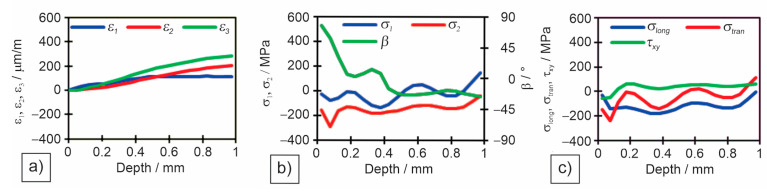
Measured residual stresses by the hole drilling method for measuring point M1—base material: (**a**) measured deformations in individual strain gauges; (**b**) measured principal stresses and angles; (**c**) measured longitudinal, transverse and shear stresses.

**Figure 15:**
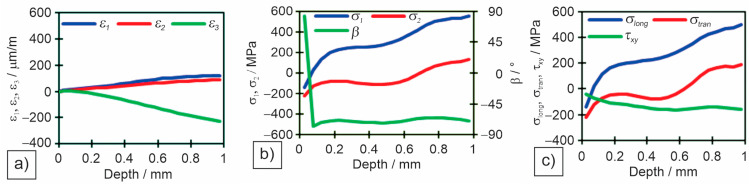
Measured residual stresses by the hole drilling method for measuring point M4—heat-affected zone: (**a**) measured deformations in individual strain gauges; (**b**) measured principal stresses and angles; (**c**) measured longitudinal, transverse and shear stresses.

**Figure 16:**
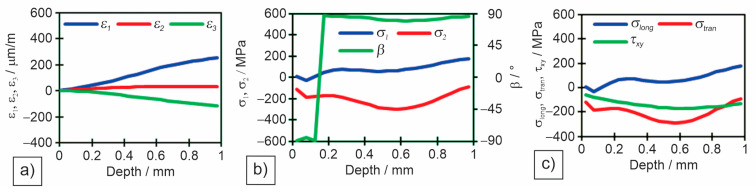
Measured residual stresses by the hole drilling method for measuring point M5—weld metal: (**a**) measured deformations in individual strain gauges; (**b**) measured principal stresses and angles; (**c**) measured longitudinal, transverse and shear stresses.

Individual measured values were read for all three measuring gauges in the rosette. Individual diagrams for measuring locations in different zones: M1—base material (Figure 14), M4—heat-affected zone (Figure 15), and M5—weld metal (Figure 16) are shown: (a) deformations (*ε*_1_, *ε*_2_, and *ε*_3_) per drilling depth, (b) principal residual stresses (*σ*_1_ and *σ*_2_) and angle of residual stresses (*β*) per drilling depth, and (c) longitudinal (*σ*_long_), transverse (*σ*_tran_) and shear (*τ*_xy_) stresses per drilling depth.

Table 9 shows the results of deformations, angle of principal stresses, principal stresses and longitudinal, transverse and shear stresses at a depth of 1 mm of drilling a hole.

The Huber–Hencky–von Mises stresses (*σ*_HH_) are the highest at the two measuring points in the HAZ (M4 and M6), as both principal stresses are tensile there. The values of *σ*_HH_ reach in M4 52%, and in M6 68% of the minimum required yield strength (YS) of the base metal. Surprisingly, the magnitude of the *σ*_HH_ in the weld metal is relatively low. This is because one of the main stresses is tensile and the other compressive, resulting in a lower *σ*_HH_ in M5, only 22% of the minimum required YS of the filler material. In all other measuring points in the base metal the *σ*_HH_ were between 105 MPa and 167 MPa, which is 10–17% of the minimum required base metals YS.

**Figure 17:**
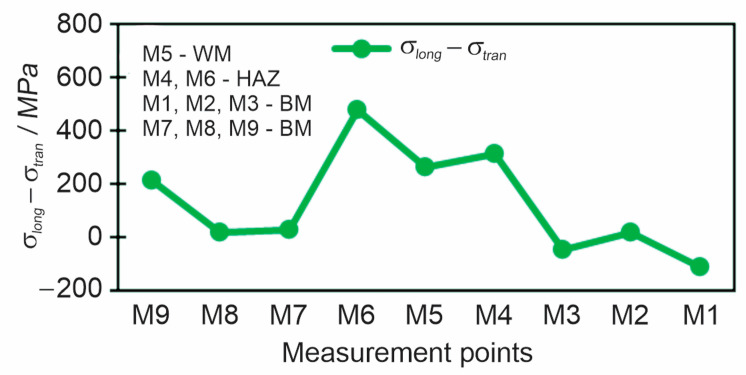
Subtraction of longitudinal and transverse residual stresses *σ*_long_–*σ*_tran_ measured by the hole drilling method at a depth of 1 mm.

The diagram in Figure 17 shows the subtraction of longitudinal and transverse stress for all nine measuring points at a drilling depth of 1 mm.

### 3.5. Microstructure

**Figure 18:**
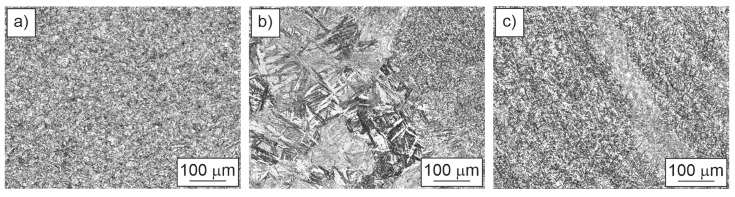
Microstructure: (**a**) base material; (**b**) HAZ; (**c**) WM—weld metal at weld toe.

Three different locations were observed: base materials, heat-affected zone and weld metal (Figure 18). Figure 18a shows the microstructure of the base material where a fine-grained martensitic microstructure can be observed. The microstructure on the left side of Figure 18b is the coarse-grained heat-affected zone, and on the right side is the weld metal. In the coarse-grained heat-affected zone, there is lath martensite with long laths, which formed during cooling of the weld joint. Figure 18c shows weld metal which has dendritic fine-grained martensitic microstructure.

## 4. Discussion

In this paper, residual stresses were measured with three different methods: the MAS, XRD, and HD methods. Each of these methods is specific and has its advantages and disadvantages. Also, when comparing these three methods, the results deviate in certain areas for several reasons. The main difference in measurements is that each method measures residual stresses at differently large areas and different depths and averages the results. The HD method covered a volume of 5.13 mm in radius, and up to 1 mm deep. MAS method measured inside a cylinder with a diameter of 25 mm and a depth of 0.5 mm, while the XRD method measured inside a cylinder with a diameter of 1 mm and a depth up to 0.010 mm under the surface. Another difference in measurements is that each of these three methods measures different types of residual stresses. Only type I can be measured with the HD method, types I and II with the HD method, and type I, II, III and IV of residual stresses with the XRD method. Also, each method provides a different result, and in order to be able to compare them, we have to harmonize them.

**Figure 19:**
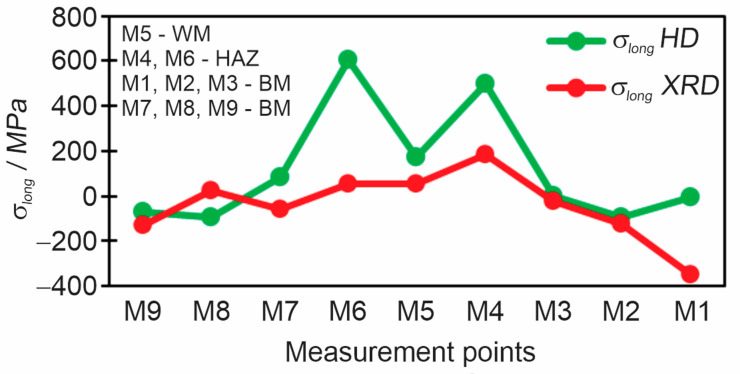
Comparison of longitudinal residual stresses between the HD and XRD methods.

**Figure 20:**
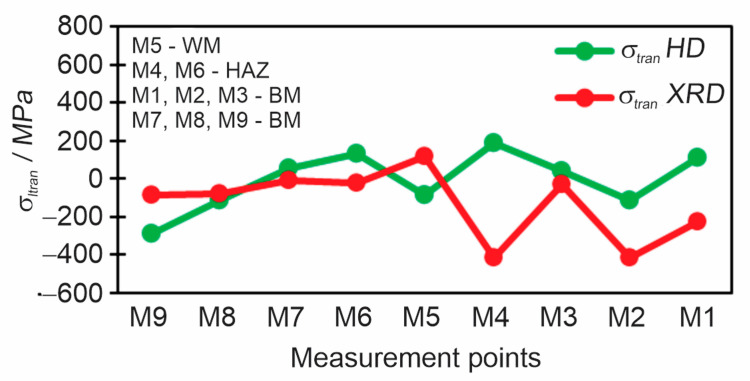
Comparison of transverse residual stresses between the HD and XRD methods.

As it is known that the XRD method gives direct residual stresses in the longitudinal and transverse directions separately, results of the HD method must be adopted to compare the HD and XRD methods as shown in Figure 19 and Figure 20. With the HD method, we obtain the principal stresses and the direction/angle of the principal stresses, and to obtain the residual stresses in the direction we want, Moore circles are used. Thereby, a direct comparison of the residual stresses *σ*_long_ (Figure 19) and *σ*_tran_ (Figure 20) is possible. The biggest differences between methods were noted in WM and HAZ because the XRD method has the disadvantage that when it encounters a coarse-grained the scattering of results is significant, as it was observed also in the case of S960QL steel. The difference is also in the type of residual stresses which both methods measure (HD—type I, XRD—type I + type II).

**Figure 21:**
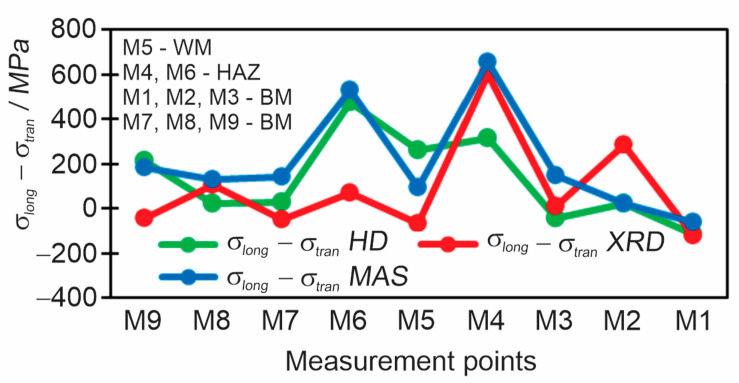
Comparison of the subtractions of longitudinal and transverse residual stresses *σ*_long_ −*σ*_tran_ between all three measurement methods.

In order to compare all three methods at once, all residual stress results should be converted to give the subtractions of longitudinal and transverse stress for each measurement point. In the MAS method, the disadvantage is that the residual stress is obtained by the subtraction of the principal stresses. By measuring the principal stresses, we can obtain in one direction the stress in the plus and the other in the minus which results in obtaining large residual stresses, which is not favorable, and makes interpretation of the results more difficult. A comparison of all three methods as a subtraction of longitudinal and transverse residual stress (*σ*_long_–*σ*_tran_) is shown in Figure 21.

Each of the applied methods covered a different volume of material. The MAS method covered a volume of *ϕ* 25 mm × 0.5 mm, the HD method covered a smaller volume of *ϕ* 5,13 mm × 1 mm, and the XRD method covered a volume of *ϕ* 1 mm × 0.01 mm. Even in the case of fine-grained, nontextured homogeneous materials and homogeneous stress fields, different methods give different results, just because due to the different volumes covered, different methods measure different types of RSs (MAS—type I + type II + type III + type IV, XRD—type I + type II, and HD only type I). Additional reasons for differences between methods are present in the case of test pieces with high stress gradients and/or microstructures containing coarse-grained areas and/or textured areas (e.g., columnar grains), because the obtained result is always an average volume for the whole volume covered: (a) in larger volumes covered, the peak stresses and stress gradients are most likely different than in a smaller volume of material; (b) in a larger volume, microstructural gradients are likely larger than in a small volume; (c) different methods exhibit different sensitivities to coarse-grained and/or textured microstructures (HD almost no sensitivity, while the accuracy of MAS and XRD decreases considerably), where not only the size exerts influence, but in the case of WRD, also predominant orientations of crystal lattices.

The biggest differences between results obtained by different methods can be observed in areas of high stress gradients and areas with coarse-grained (HAZ) and coarse-grained textured microstructures (weld metal), as shown in Figure 21. Particularly distinct are the differences at the measuring point M6. M6 was located in the HAZ, which is a quite narrow zone. The MAS method covered an area significantly wider than the width of HAZ. Therefore, in spite the center of the probe was positioned in the HAZ, most of the volume covered was weld metal and base metal, while the HAZ represented only a minor fraction. Consequently, the result was predominantly influenced by weld metal and base metal. The HD method covered a smaller volume, 5.13 mm in diameter, but still the covered volume contained considerable fractions of weld metal and base metal. Only in the case of the XRD method, which covered a diameter of 1 mm, the whole volume covered was located inside the HAZ. Considering this and the reasons explained in the preceding paragraph, the results in areas like HAZ must inevitably differ, and it can also be expected differences to be substantial in some cases. Nevertheless, the most likely reason for the exceptionally large differences in point M6 are deviations of XRD measurements. In other measuring points, the system calculated deviations ±10–17 MPa, but in M6 deviations were much larger. Therefore, in the measuring point, measurements were repeated three times, but deviations were always extraordinarily large: ±65 MPa, ±68 MPa, and ±72 MPa. So, the XRD result for M6 is unreliable, and the RSs are most likely much closer to values measured by the MAS and HD methods. The large deviations of XRD results in M6 are most likely a consequence of the microstructure, which however cannot be confirmed by metallographic examination, because later a hole was drilled there for HD method measurements.

## 5. Conclusions

The superposition of the welding residual stresses with working stresses caused by operating condition can lead to extending defects or crack initiation and their propagation up to the point where the structure needs to be repaired or even withdrawn from use. Therefore, knowing the residual stresses is very important for design and prediction of the life of welded structures, in this paper, residual stresses were measured using three different methods on V-shaped butt weld joints. These methods were the magnetic method, the X-ray diffraction method, and the hole drilling method. Their main advantages and disadvantages are summarized in Table 10. At the end, results of the residual stresses were compared. The highest tensile residual stresses were obtained in the HAZ and the weld metal. In other areas (base material), tensile or compressive residual stresses appeared. When attempting to compare the residual stress measurements in the same measuring point, numerous points need to be taken into account: (a) the HD method determines the principal stresses and the direction-angle of the principal stresses, the XRD method gives direct residual stresses in the longitudinal and transverse directions separately, and the result of the MAS method is the subtraction of principal stresses *σ*_1_–*σ*_2_ and the directions of principal stresses are known; (b) the volumes of material and depths where residual stresses are averaged during measurement differ considerably; (c) what types of stresses each method measures (type I, type II, type III, and type IV); and (d) microstructural changes in HAZ and newly formed coarse columnar grains in weld metal, which both significantly decrease the reliability, especially in the case of the XRD method. Consequently, comparison of the methods is not so easy. To make results as comparable as possible, subtractions of principal stresses *σ*_1_–*σ*_2_ must also be calculated for HD and XRD, whereas for the XRD method, the stress values in directions of principal stresses must be determined first by the use of Moore circles. Nevertheless, due to the reasons summarized in points (a) to (d), the values can still differ substantially. In future work, we plan to use the same three methods of residual stress measurement, for mutual comparison, on a welded joint of high-strength anti-ballistic steel.

## Figures and Tables

**Table 1:** Chemical composition of the S960QL steel plate.

Element—Mass Fraction/wt. %				
C—0.174	B—0.0027	Cr—0.623	N—0.0015	Ti—0.002
Si—0.297	P—0.007	Cu—0.043	Nb—0.027	V—0.002
Mn—1.070	S—0.0017	Mo—0.612	Ni—0.052	Zr—0.001

**Table 2:** Basic mechanical properties of the S960QL steel plate.

Thickness/mm	*R*_p0.2_/MPa	*R*_m_/MPa	*A*/%	*KV*/J
15.0	1014	1049	13	150, 153, 156 (−40 °C, long.)
	1024	1060	13	45, 43, 49 (−40 °C, tran.)

**Table 3:** Chemical composition of filler material.

Element—Mass Fraction/wt. %												
C	Si	Mn	P	S	Cr	Mo	Ni	V	Cu	Ti	Al	Zr
0.09	0.78	1.79	0.008	0.008	0.36	0.70	2.15	<0.01	0.04	0.06	<0.01	<0.01

**Table 4:** Welding parameters.

Welding Process: MAG − 135 According to EN ISO 4063:23; Filler Wire Diameter *ϕ* = 1 mm; Current DC+							
Run	1	2	3	4	5	6	7
Welding current/A	208	230	240	240	252	258	266
Welding voltage/V	27.5	26.5	26.8	26.8	28	29	30
Wire speed/m∙min^−1^	10.7	11.5	12	12	14	14.3	16
Travel speed/cm∙min^−1^	40	38	38	38	35	34	30
Heat input/kJ∙cm^−1^	6.8	7.6	8.1	8.1	9.6	10.5	12.7
Shielding gas/l∙min^−1^	15	15	15	15	15	15	15
Preheating/°C	100	-	-	-	-	-	-
Max. interpass *T*/°C	-	150	150	150	150	150	150

**Table 5:** Locations of residual stress measurements.

Location	*X*/mm	*Y*/mm	Area in the Weld Joint *
M1	350	−120	BM
M2	350	−70	BM
M3	350	−30	BM
M4	350	−14	HAZ
M5	350	0	WM
M6	350	+14	HAZ
M7	350	+30	BM
M8	350	+70	BM
M9	350	+120	BM

* BM = base material; HAZ = heat-affected zone; WM = weld metal.

**Table 6:** Results of the tensile test.

Specimen	Measured Ultimate Tensile Strength *R*_m_/MPa	Reduction in Area *Z*/%	Ultimate Tensile Strength of the Base Metal *R*_mp_/MPa
TT-1	1025	39	980–1150 *
TT-2	1038	33	980–1150 *

* Required by EN 10025-6:2019+A1:2023 [45].

**Table 7:** Results of the magnetic method.

Measurement Point		Signal *σ*_1_–*σ*_2_/mV	Subtraction of Principal Stresses *σ*_1_–*σ*_2_/MPa
M9	BM	57.6	185
M8	BM	39.7	127
M7	BM	44.0	141
M6	HAZ	152.1	531
M5	WM	29.4	93
M4	HAZ	181.4	658
M3	BM	46.6	149
M2	BM	7.9	25
M1	BM	−18.6	−59

**Table 8:** Results of residual stresses measured by the X-ray diffraction method.

Measurement Point		Residual Stresses in Longitudinal Direction *σ*_long_/MPa	Residual Stresses in Transverse Direction *σ*_tran_/MPa	Subtraction of Long. and Tran. Residual Stresses *σ*_long_*–σ*_tran_/MPa
M9	BM	−130	−85	−45
M8	BM	25	−80	105
M7	BM	−57	−7	−50
M6	HAZ	52	−19	71
M5	WM	53	119	−66
M4	HAZ	186	−415	601
M3	BM	−22	−31	9
M2	BM	−125	−413	288
M1	BM	−349	−228	−121

**Table 9:** Results of measurement residual stresses by the hole drilling method at a depth of 1 mm.

Measurement Point		Principal Stress *σ*_1_/MPa	Principal Stress *σ*_2_/MPa	Huber–Hencky–Von Mises Stresses *σ*_HH_/MPa	Angle of Principal Stresses *β*/°	Longitudinal Stress *σ*_long_/MPa	Transverse Stress *σ*_tran_/MPa	Shear Stress *τ*_xy_/MPa	Subtraction of Long. and Tran. Stresses *σ*_long_*–σ*_tran_/MPa
M1	BM	−41	147	153	−26.2	−4	110	57	−114
M2	BM	−162	−42	167	49.8	−92	−112	−10	20
M3	BM	−55	98	112	−36.3	−1	44	22	−45
M4	HAZ	136	553	569	−69.4	501	188	−156	313
M5	WM	−90	176	198	85.6	175	−88	−132	263
M6	HAZ	93	644	651	−74.8	607	131	−238	476
M7	BM	41	97	105	59.5	82	55	−13	27
M8	BM	−162	−42	167	49.8	−92	−112	−10	20
M9	BM	−339	−19	153	65.9	−72	−285	−106	213

**Table 10:** Advantages and disadvantages of residual stress measurement methods.

Method	Type of Residual Stress	Advantages of the Method	Disadvantages of the Method
MAS	type I + type II + type III + type IV	Measuring RSs inside a cylinder of *ϕ* 25 mm × 0.5 mm. Easy-to-implement method in the field. The most economically acceptable method.	Difficult interpretation of the results *σ*_long_–*σ*_tran_ due to high differences between *σ*_long_ and *σ*_tran_. If the HAZ and WM areas are small, the diameter can hardly hit the desired area.
XRD	type I + type II	Measuring RSs at a depth of 0.001 mm under the surface inside a cylinder with a diameter of 1 mm. The desired area/point of measurement can be hit easily and precisely. The method gives direct results *σ*_long_ and *σ*_tran_.	The results depend a lot on the quality of the electropolishing. Coarse columnar grain causes significant scattering of results.
HD	type I	Standardized method. Measuring RSs at a depth up to several millimeters inside a cylinder with a diameter of several millimeters, depending on the applied rosette type. The method gives the results of deformations, principal stresses, and the angle of the principal stresses. Results can be obtained at different depths depending on the drilling depth and drilling step. Using Moore’s circles, the results can be transformed into *σ*_long_ and *σ*_tran_.	Method covers only macro deformations (type I). Expensive and demanding method due to the purchase of one-time use rosettes and rosette gluing.

## Data Availability

The original contributions presented in this study are included in the article. Further inquiries can be directed to the corresponding author.
